# Cement leakage in a symptomatic vertebral hemangioma: a case report and review of the literature

**DOI:** 10.1186/1757-1626-2-7148

**Published:** 2009-05-06

**Authors:** Dimitrios S Evangelopoulos, Panos Kontovazenitis, Konstantinos Kokkinis, Michalis Glynos, Dimitrios S Korres, George Sapkas

**Affiliations:** 1 1^st^ Department of Orthopaedics, University of Athens, KAT HospitalNikis 2 Street, Athens, 14561Greece; 2 Radiology Department, KAT HospitalNikis 2 Street, Athens, 14561Greece; 3 Radiology Department, Metropolitan HospitalEthn. Makariou 9 & El. Venizelou 1 Street, Athens, 18547Greece; 4 Department of Orthopaedics, Spine Unit, Metropolitan HospitalEthn. Makariou 9 & El. Venizelou 1 Street, Athens, 18547Greece

## Abstract

We present the case of a 50-year-old male with consistent back pain, not resolving with conservative treatment. Plain radiograms demonstrated a lytic lesion at the level of the 8^th^ thoracic vertebra. Thorough examination with computerized tomography and magnetic resonance imaging revealed a hemangioma extending to the posterior third of the vertebral body, compressing the spinal cord at the level of 8^th^ thoracic vertebra. A percutaneous vertebroplasty was performed. The post-operative computerized tomography scan demonstrated cement leakage. After thorough cement removal combined with extensive decompression and posterior stabilization, the patient reported gradual improvement of his symptoms and was able to return successfully to his work a few months later.

## Introduction

Hemangiomas represent benign tumors, originally described by Virchow in 1867. These common lesions, found in 10% to 12% of autopsy specimens, are defined as vascular spaces lined with endothelial cells. They are usually detected in the fourth or fifth decade of life and are twice as likely to be present in women as in men [1]. Most of them remain clinically silent and are usually detected incidentally during random x-ray examinations. Occurrence may be multiple or single at one or more different levels, mainly in the thoracic and lumbar spine. They can be located on the periosteal surface, within the cortex, or within the medullary canal. Involvement of posterior elements is indicative of an aggressive tumor. Although asymptomatic, these lesions may lead to: (i) pain, (ii) neurological compromise due to hypertrophy of the posterior cortex of the vertebral body, extension to the epidural space, occurrence of a compression fracture or hemorrhage and (iii) symptomatic fracture [2].

Microscopically, hemangiomas are distinguished as: capillary, cavernous, arteriovenous and venous with the first two groups being the most prevalent in osseous tissue. Clinically, they are subdivided into three main subcategories: the most common asymptomatic hemangioma, the compressive vertebral hemangioma and the rare symptomatic hemangioma [3].

Diagnosis is mainly based on CT and MRI findings. In CT-scans, hemangioma is presented with a characteristic “polka dot” appearance, while in MRI it is detectable in both T1 and T2 sequences, with T2 being the most diagnostic [4].

## Case presentation

We describe the case of a 50-year-old Greek Caucasian male with a free medical history, who visited his primary care physician for back pain. Since initial conservative treatment did not resolve his symptoms, he was referred to our department.

**Figures 1 and 2.**
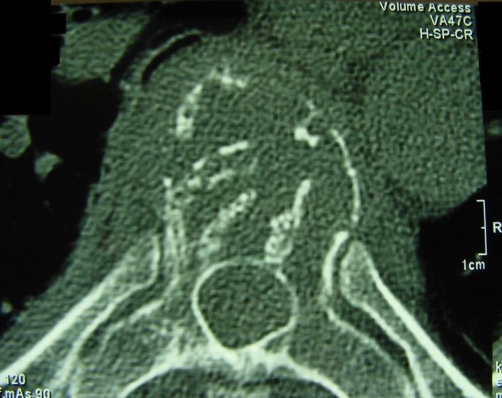

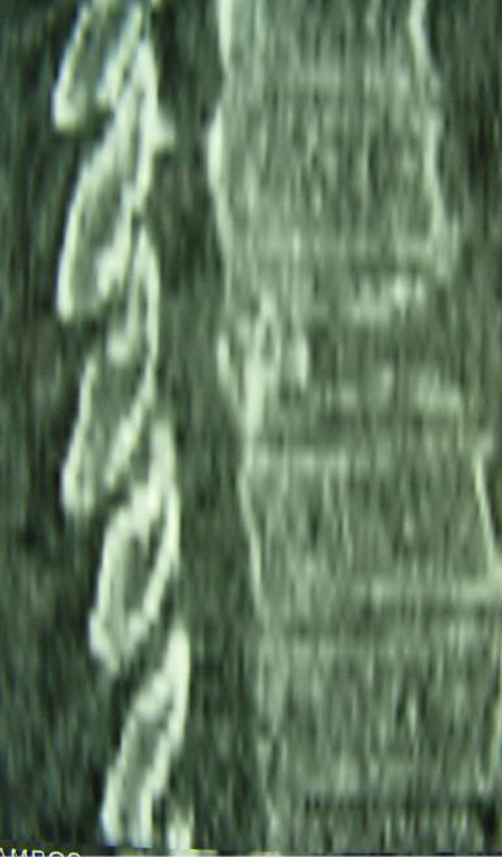
Transaxial and MPR Sagittal CT images reveal the partially collapsed vertebra with typical findings of an hemangioma (thickening of some of the vertical trabeculae with dissolution of horizontal trabeculae) involving both the vertebral body and the posterior elements. Note the small paraspinal mass and focal cortical erosions.

On examination, he complained of consistent back pain. Clinical examination did not reveal increased sensitivity or any neurological deficit. Plain radiograms and CT scan of the thoracic spine demonstrated a hemangioma at the level of T8 vertebra (Figures 1 and 2).

**Figures 3 and 4.**
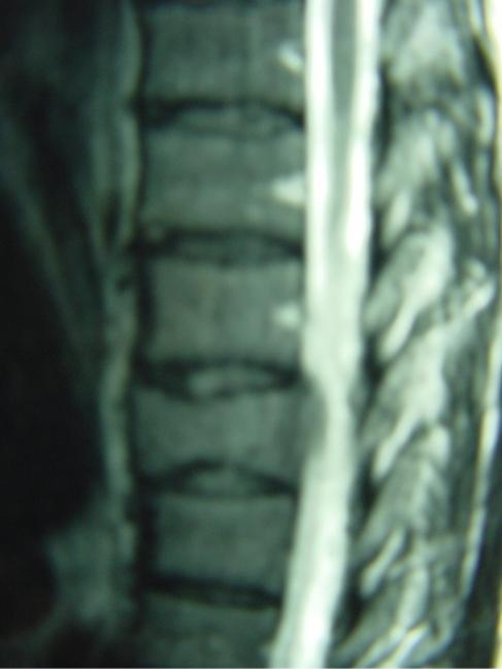

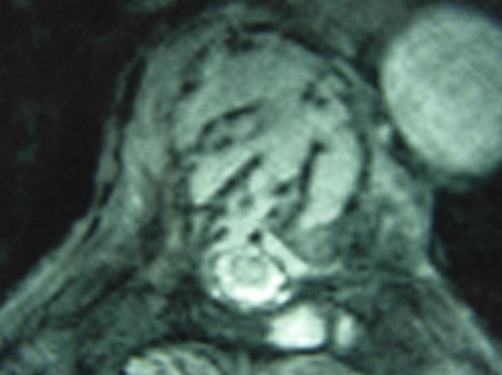
MRI Sagittal and Transverse T2-W images reveal the extraosseous extension in the anterior epidural space compressing the spinal cord.

On a following examination, the new CT scan indicated a fracture of T8 while MRI revealed an unspecific image of the paravertebral space, the fracture of the T8 vertebra and spinal stenosis at the same level (Figures 3 and 4).

**Figure 5.**
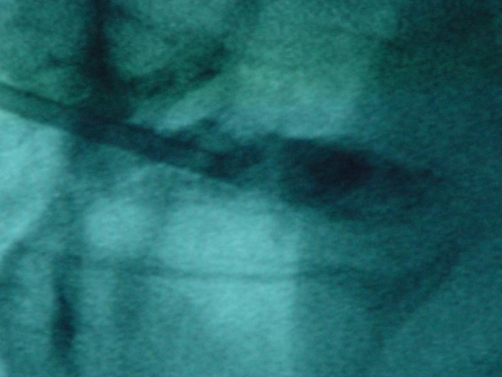
Percutaneous Vertebroplasty.

**Figure 6.**
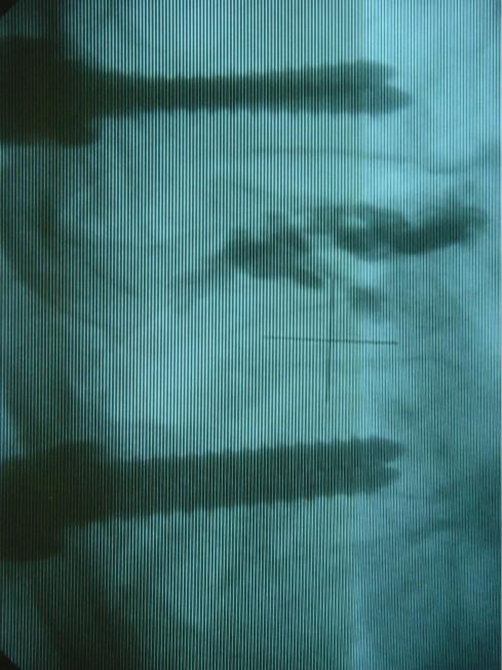
Th8 laminectomy and posterior stabilization from Th7 to Th9 after cement removal.

**Figure 7.**
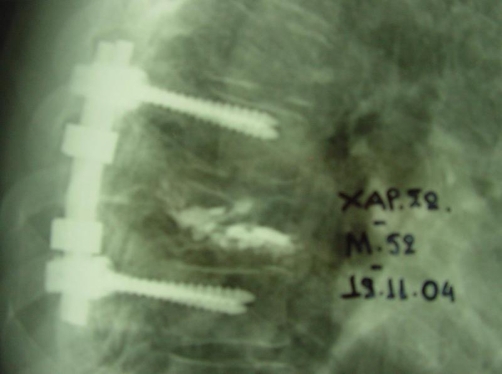
Final post-op x-ray.

**Figures 8 and 9.**
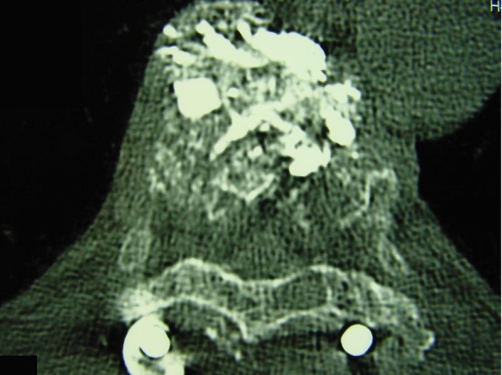

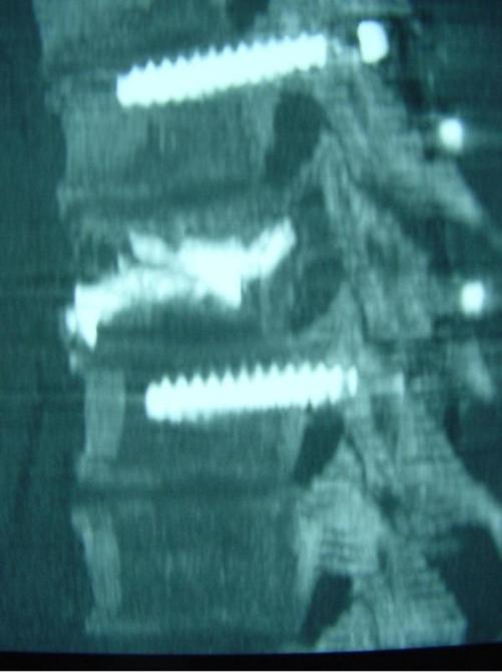
Axial CT and 3D CT sagittal postoperative images.

In order to retain vertebral height and relieve our patient from his symptoms, a percutaneous vertebroplasty at the level of T8 was performed (Figure 5). Post operatively, pain was still present and new x-rays and CT scan revealed cement leakage. Immediate cement removal and extensive laminectomy were performed, combined with posterior stabilization from T7 to T9, to further stabilize the area (Figures 6-9). After surgery, our patient’s symptoms gradually resolved. He was mobilized and returned to work a few months later.

## Discussion

Hemangiomas represent benign, silent lesions that usually remain undetected. Symptoms arise only when spinal cord compression occurs.

Treatment of such symptomatic lesions includes several options. Embolization, radiotherapy and ethanol perfusion of the hemangioma present good results and can be used therapeutically to reduce the vascularity of the region and diminish bleeding. Laminectomy can also be performed, in case of spinal compression [5]. Percutaneous vertebroplasty is a well documented treatment option for asymptomatic hemangiomas. Deramond et al. [6] have reported the treatment of an aggressive spinal hemangioma with this technique. The concept of the authors was to inject methyl methacrylate polymer under fluoroscopic control into the osteolytic vertebral body. Cement polymerization enhanced bone mechanical properties, thus preventing vertebral collapse. Because of its high success rate, percutaneous vertebroplasty’s indications gradually evolved comprising most metastatic lesions as well as osteoporotic compression fractures. Despite of its excellent results and minimal invasiveness, this technique may be the cause of several complications, such as local infection, pedicle fracture, pulmonary embolism, as well as cement leakage. The latter represents one of the most serious complications since leakage of cement in the spinal canal may lead to permanent neurological deficit requiring surgical decompression.

Hochegger et al. [7] reported a rare complication where PMMA failed to stabilize the unstable vertebral fracture, leading to symptomatic spinal canal stenosis four months later requiring surgical decompression. Chan-Chin Yu et al. [8] reported in 2007 the epidural and neuroforaminal extravasation of polymethylmethacrylate after percutaneous vertebroplasty in the thoracic spine requiring surgical intervention. In the same year, an intradural cement leakage requiring extensive laminectomy was described, by Sabuncuoglu H. et al. [9]. Additionally, Jung-Kuei et al. [10] presented a symptomatic combined extraforaminal and intradiscal cement leakage requiring a L2-L3 intertransverse process approach with removal of extraforaminal leaked cement and posterior instrumentation from L3-L5 and posterior fusion.

These serious complications have troubled most of the surgeons dealing with the technique. In an effort to decrease the possible risk of spinal canal compression, Wenger M et al. [11] proposed the prophylactic fenestration of the spinal canal in all patients allowing thus the continuous evaluation of neural structures. Moreover, it makes possible the immediate removal of cement prior to nerve damage from heat and compression. Of course, in this way, the authors convert percutaneous vertebroplasty into an open procedure with concomitant increased morbidity. The performance of a transosseous venography prior to cement injection has also been proposed as a helpful measure in preventing cement leakage. Nevertheless, for some authors this technique has not been helpful with regard to the prevention of cement spillage and therefore has been abandoned [11]. Since nowadays vertebroplasty has become a widespread technique, several other methods have been proposed for minimizing the risk of cement leakage: the use of tantalum-impregnated polymethylmethacrylate in very small (2-cc) amounts [12], the use of cement in a doughy rather than liquid consistency [13], injection with minimal-constant pressure [14], the placement of large needles well anteriorly into the vertebral body through both pedicles and finally the new technique of balloon kyphoplasty [15] which effectively prevents posterior extrusion of cement while allowing at the same time some expansion of the collapsed vertebra.

Nevertheless, in our case despite of the use of special cement, the anteriorly placed needles, the initial injection of small amount of cement and the continuous radiological evaluation, a cement leakage occurred.

## Conclusions

Percutaneous vertebroplasty is a well documented treatment option for asymptomatic hemangiomas. Nevertheless, the possibility of cement leakage should always be taken into consideration when dealing with such a minimal invasive technique and extreme consciousness and continuous radiographic evaluation are mandatory in order to be able to detect cement leakage and proceed to immediate decompression and cement removal.

## Abbreviations

CT: Computer tomography
MRI: Magnetic resonance imaging
PMMA: Polymethylmethacrylate
